# Introducing an indoor object classification dataset including sparse point clouds from mmWave radar

**DOI:** 10.1038/s41597-024-03678-2

**Published:** 2024-08-03

**Authors:** Panagiotis Kasnesis, Christos Chatzigeorgiou, Vasileios Doulgerakis, Dimitris Uzunidis, Evangelos Margaritis, Charalampos Z. Patrikakis, Stelios A. Mitilineos

**Affiliations:** 1 University of West Attica, Department of Electrical and Electronic Engineering, Egaleo, 12241 Greece; 2 ThinGenious PC, Marousi, 15125 Greece

**Keywords:** Electrical and electronic engineering, Research data

## Abstract

This document introduces the RadIOCD, which is a dataset that contains sparse point cloud representations of indoor objects, collected by subjects wearing a commercial off-the-shelf mmWave radar. In particular, RadIOCD includes the recordings of 10 volunteers moving towards 5 different objects (i.e., backpack, chair, desk, human, and wall), placed in 3 different environments. RadIOCD includes sparse 3D point cloud data, together with their doppler velocity and intensity provided by the mmWave radar. A total of 5,776 files are available, with each one having an approximate duration of 8s. The scope of RadIOCD is the availability of data for the recognition of objects solely recorded by the mmWave radar, to be used in applications were the vision-based classification is cumbersome though critical (e.g., in search and rescue operation where there is smoke inside a building). Furthermore, we showcase that this dataset after being segmented into 76,821 samples contains enough data to apply Machine Learning-based techniques, ensuring that they could generalize in different environments and “unseen“ subjects.

## Background & Summary

Object detection and identification is very important for a broad gamut of smart home/office, safety and security applications, such as occupancy detection, personalized heating and cooling, natural light adjustment, etc. In prior art, several computer vision-based techniques have shown a good performance when given a clear field of view^1,2^, however, when it comes to scenarios with adverse conditions (e.g., a room full of smoke due to fire), such as Search and Rescue (SaR) operations, camera’s sensing capabilities degrade. An alternative technique is that of processing Wi-Fi signal. For example, Zou *et al*.^3^ utilized the variations in ambient Wi-Fi signals to recognize people based on their gait. However, the proposed method requires a separate transmitter and receiver, and its performance decreased significantly in cases when users walk between the transmitter and receiver, and, more importantly are incapable of simultaneously tracking and identifying multiple people in the same scene^4^.

A solution to this issue are radar sensors based on millimeter waves (mmWave) that are capable of remotely sensing obstacles and objects, offering the capacity for an augmented sensory perception to robots, autonomous vehicles and even humans (e.g., visually impaired). Radars are capable of operating at low frequencies as below 10 GHz up to as high as 100 GHz, resulting to ameliorated levels of scenery resolution and increased degrees of freedom based on the specific requirements of each application^5^. Moreover, mmWave radars are a much cheaper technology than LiDARs which are, furthermore, not very robust when it comes to operating under adverse conditions^6^, leading to the common use of mmWave radars in automotive applications and for multiple object tracking^4,7,8^.

When it comes to radar object tracking and identification there do exist two main data formats for analysis: a) processing micro-Doppler (*μ*D) signatures (i.e., *μ*D spectrograms) and b) processing point-clouds. The first approach usually involves deep learning algorithms (i.e., Convolutional Neural Networks) applied to the radar-generated *μ*D spectrograms^9,10^. However, even though they are accurate, these algorithms deal with non-sparse radar range-azimuth-Doppler maps that require a large communication bandwidth to transfer the raw radar signals from the radar board to the processing device, preventing their implementation on low-cost embedded boards used for edge computing^11^. When it comes to datasets including radar generated point clouds and consequently approaches on processing them, unfortunately, there exist few works, since, unlike LiDAR generated point clouds, radar point clouds are sparse making the identification task more challenging.

To the best of our knowledge, Radar-based Indoor Object Classification Dataset (RadIOCD) is the first publicly available dataset including point clouds representing indoor items generated by an mmWave radar wearable device. RadIOCD could lead to the development of applications detecting hazardous, moving or approaching objects that may be either covered with smoke, fog etc.; these application are well-suited for users who are visually impaired due to their sensory capabilities or the environmental conditions, e.g., first responders operating in an environment covered with smoke^12^. In contrast to existing radar-based point cloud datasets captured in indoor scenarios where the radars are installed in the ambience^13–15^ (e.g., placed at a wall, or on a furniture) or on moving robots^16–18^, in our dataset the mmWave device was attached to a human’s belt, thus essentially turning it to a wearable sensor and enabling its application in unknown environments^19^ without the need of pre-scanning the area by a robot. When it comes to search and rescue operations every second could be life critical, thus, the fast scanning of the environment is vital.

While the advantages of using an mmWave radar as a wearable device have been highlighted in research works for developing applications to assist visually impaired people, their use is limited for binary classification tasks, predicting whether the user will collide with an object and the built datasets are not publicly available^19–21^. RadIOCD contains a total of 5,776 examples, generated by 10 participants that moved towards 5 objects of interest, carrying a custom prototype device equipped with the mmWave radar. We advocate that RadIOCD could lead to the design and development of innovative machine learning-based solutions and open the path for the creation of similar datasets and applications (e.g., 3D full body pose estimation^22^).

## Methods

### Participants and ethical requirements

The 10 subjects that participated in the data collection process were members of the RESCUER^23^ consortium, with their identities being pseudo-anonymized using number IDs instead of names, or emails. Table 1 presents their personal details, with M and F standing for masculine and feminine, respectively. Their age ranged from 24 to 46 years old, their height from 170 to 183 cm and their weight from 52 to 115 Kg. This study was approved by the Research Ethics Committee of the University of the West Attica (Approval No. 50150, 28/06/2021). It should be noted, also, that all participants signed a written informed consent for the collected data to be published.

**Table 1:** Anthropometric data, age, weight, height and sex of volunteers.

Participant ID	Gender (M/F)	Height (cm)	Weight (Kg)	Age (Years)
1	M	178	71	38
2	M	172	115	24
3	F	170	52	29
4	M	181	95	28
5	F	170	81	37
6	M	183	82	46
7	M	180	80	36
8	M	182	80	46
9	M	171	100	37
10	M	183	77	37

### Acquisition setup

The RadIOCD includes point cloud data recorded with an IWR1443BOOST^24^ radar sensor. Point clouds are generated directly by the board using the following pipeline^25^; the receiver antennas (Rx) of the board receive the reflected radar signal, which is mixed with the transmitted signal in order to generate the intermediate frequency signal, which is then delivered to the Analog-to-Digital Converter (ADC). The ADC converts its analog input to digital samples for further digital processing with 2D Fast Fourier Transformation (FFT); more specifically, the range-FFT is applied to estimate the targets’ ranges while the Doppler-FFT is utilized to obtain the targets’ Doppler velocities. The next step is the Cell-Averaging Constant False Alarm Rate (CA-CFAR) algorithm to eliminate the false alarms and finally estimate the Angle of Arrival (AoA) exploiting the 3 Tx and 4 Rx configuration. After AoA estimation, a sparse point cloud is generated, with each point providing values regarding their X, Y and Z coordinates and the radial velocity.

The IWR1443Boost sensor was configured to use all three transmitter antennas and all four receiver antennas in order to generate 3D point cloud data. The sampling rate was set to 10Hz, with range resolution equal to 0.044m, maximum unambiguous range of 8.00m, maximum radial velocity equal to 2.35m/s and radial velocity resolution of 0.3m/s. The configuration details are, also, tabulated in Table 2

**Table 2:** Configuration values of the AWR1443BOOST radar.

Variable	Value
Frequency	77-81 GHz
Bandwidth	4 GHz
Frequency slope	70 GHz/ms
Ramp duration	57.14 *μ*s
Chirps per frame	16
Number of ADC samples	4558
Number of TX antennas	3
Number of RX antennas	4
Azimuth resolution	15 degrees + elevation
Range resolution	0.044 m
Maximum range	8.00 m
Maximum radial velocity	2.35 m/s
Radial velocity resolution	0.3 m/s
Frame duration	100 ms
Range detection threshold	15 db
Frame rate	10 Hz

**Fig. 1:**
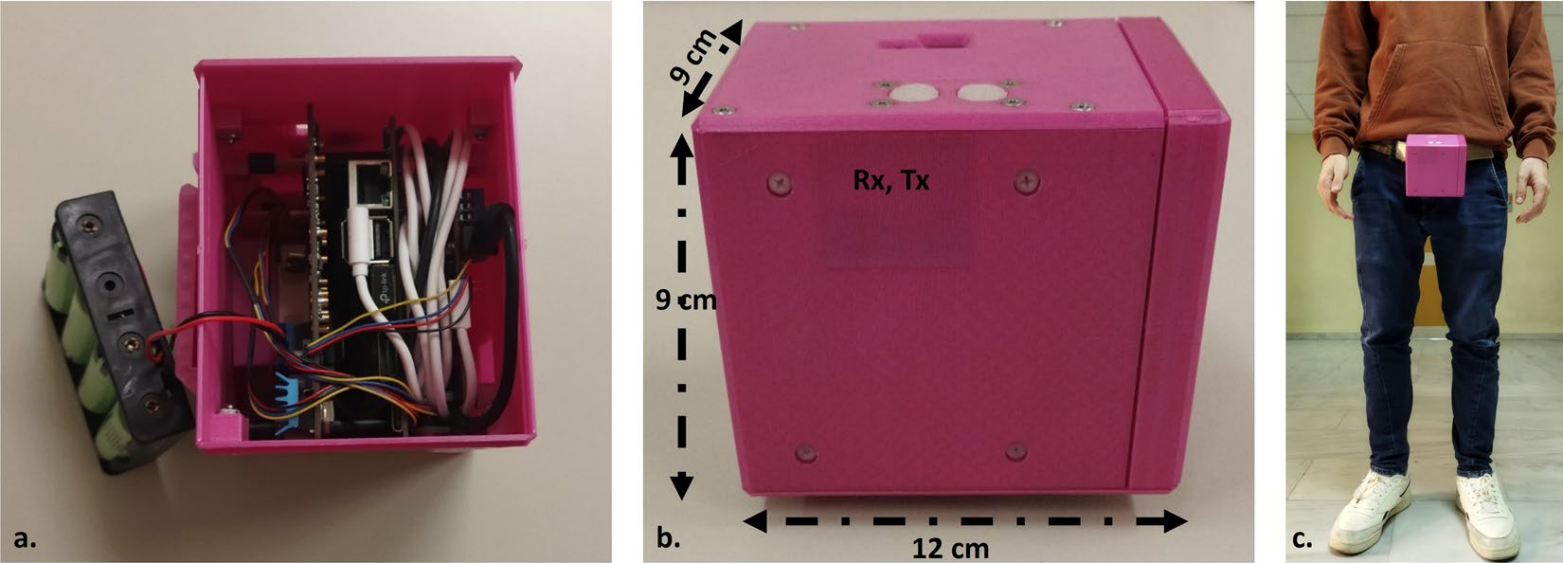
The developed device assembled by a Jetson Nano, the IWR1443BOOST radar, the CHM and a set of batteries shown at the left (**a**). At the middle (**b**) the dimensions of the device are displayed along with dark pink area composed by a very thin Polylactic acid layer (1mm) allowing the transmission and reception of the mmWave (Rx, Tx area). The device placement is illustrated at the right (**c**).

The developed prototype device is comprised by a Jetson Nano^26^ Single-Board Computer (SBC), an IWR1443BOOST^24^ radar, a CHM (Custom Host Module), which communicates with the SBC via the Universal Asynchronous Receiver-Transmitter (UART) hardware protocol and a set of batteries. The assembled prototype and its dimensions are shown in Fig. 1a,b The generated sparse point clouds were recorded on an SD card and as each volunteer finished performing the designed data collection routine, we downloaded the data to the computer for further data processing. More specifically, we developed an MQTT-based data recording application running on a smartphone, where the broker and a subscriber run on the included Jetson Nano and as soon as they receive a particular message they start or stop recording.

**Fig. 2:**
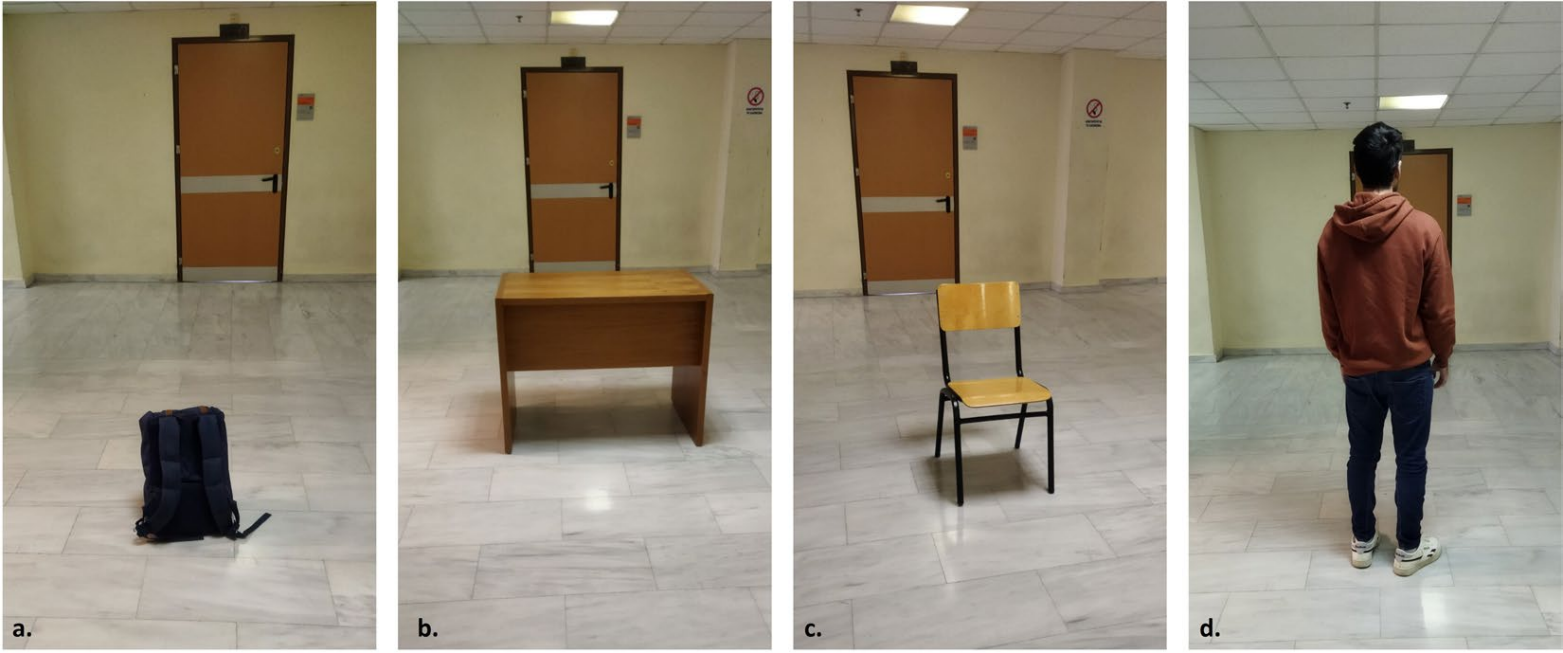
The selected indoor objects for training the classification module: (**a**) backpack, (**b**) desk, (**c**) chair and (**d**) human. The 5^*th*^ class, i.e. wall, for this particular environment is shown at the background of each figure.

**Fig. 3:**
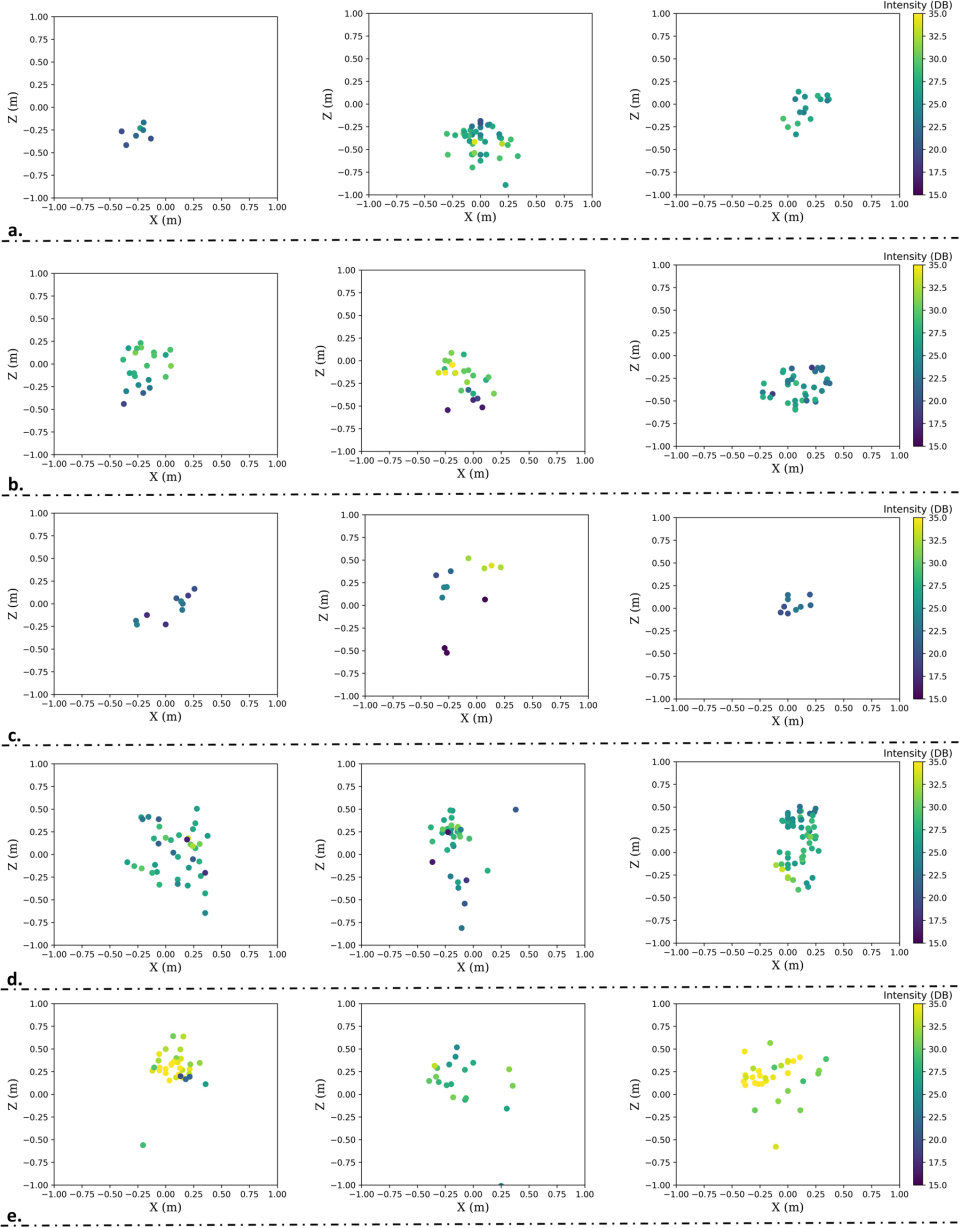
Visualization of some example point clouds (**a** backpack, **b** chair, **c** desk, **d** human and **e** wall), including their X, Z and intensity values, while only values less than 2.5 and more than 1.0 meters away from the radar are considered (Y coordinate).

As a final step, we selected 5 target objects (i.e., classes) ranging in shapes and sizes to create the dataset, which, typically, are commonly encountered in indoor environments within buildings, such as offices. In particular, we selected: a. backpack, b. desk, c. chair and d. human, and e. wall. Figure 2 displays the objects of interest, while Table 3 presents their dimensions. It should be noted that the selected desk and chair objects do not have a homogeneous mass distribution (e.g., there are a lot of gaps between their legs), so in some examples the radar was not able to receive any reflections. This is evident by visualizing the X and Z coordinates of the acquired point clouds as shown in Fig. 3. For example, Fig. 3c. displays some data collected from the desk, where for the case of the left and right example only its upper part provided a reflection, while in the middle one the radar received, also, a reflection by one of the two legs. Moreover, the backpack was, also, in some cases not captured by the radar due to its small size (i.e., only 50 cm tall).

**Table 3:** Average dimensions of the selected objects of interest.

Object	X (width in cm)	Y (depth in cm)	Z (height in cm)
Backpack	30	11	50
Desk	76	60	76
Chair	36	45	80
Human	35–60	25–50	170–183
Wall	>200	NA	>250

### Acquisition protocol

**Fig. 4:**
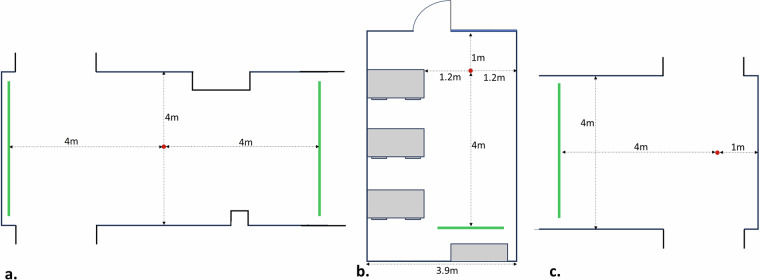
Floor plans of the selected 3 environments. The red dots demonstrate where the center of the target object was placed, and the green lines show the range of the starting position of the participants. Environment 1 (**a**) is a hallway, environment 2 (**b**) an office, and environment 3 (**c**) is, also, a hallway.

The recording took place in the premises of the University of West Attica, and more particular in three different areas, namely environment 1 (hallway), environment 2 (office) and environment 3 (hallway). While in environment 1 the objects were placed at least 2 meters away from the surrounding walls, in environment 2 and environment 3, the center of the objects was only 1 meter away from the walls or surrounding objects in an attempt to make the recognition process more challenging (Fig. 4). After a few iterations, the objects were rotated to capture several angles (front, back, side), ensuring the algorithm’s robustness to different fields of view.

The device including the radar, the NVIDIA Jetson Nano and the CHM was mounted on the belt of the user, having an orientation towards the ground (this was not fixed to certain degrees to ensure the algorithm’s generalizability to unseen orientations). Figure 1c illustrates the placement of the device. During the data collection process each participant was asked to move slowly (around 0.5 m/s) towards a selected object, starting from around 4-5 meters away from the object of interest. Each participant also carried a mobile device in his/her hand, which was used by the subjects to send triggering (start or stop) messages to the MQTT broker. It should be noted that the participants were asked to terminate the recording few centimeters before reaching the target object. Moreover, a person from the development team always supervised the whole process.

## Data Records

**Fig. 5:**
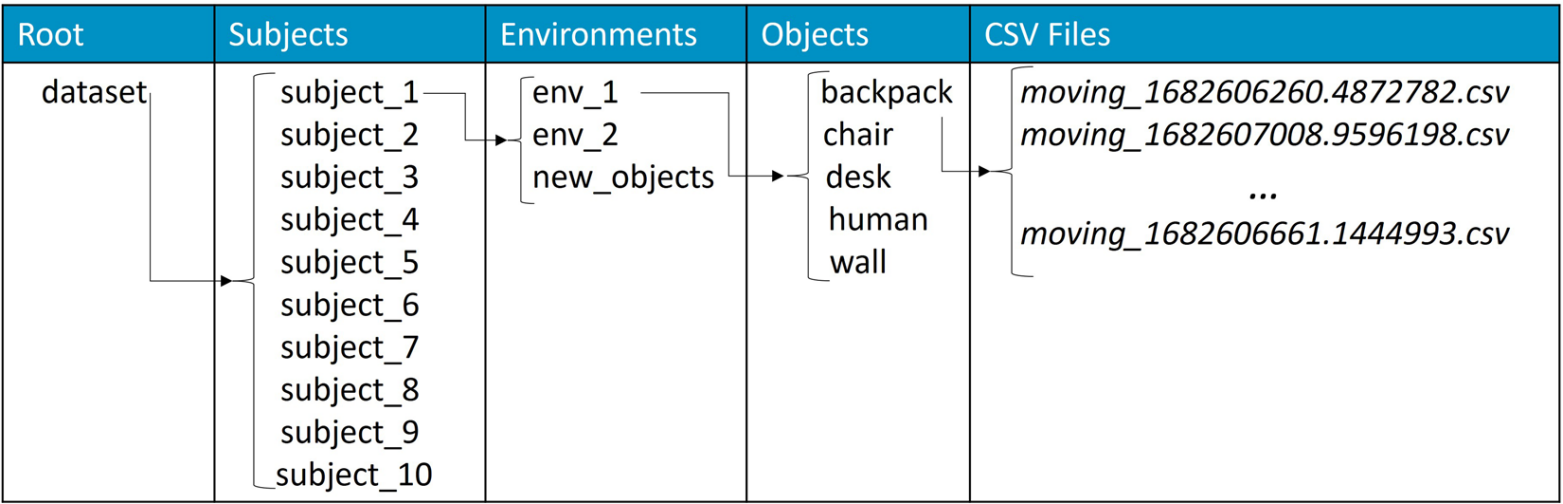
Data organization of RadIOCD. The “subject_ID” folders are included in the root directory, “dataset”. The subject-dependent folders are split into environments, which are further divided into the types of the recorded objects. Each of these contain the timestamped CSV files containing the sparse point cloud data.

All raw data files exported from the mmWave radar were stored as CSV files in the Jetson Nano platform and have been uploaded to Zenodo^27^, where a total of 5,776 files are available (the total number of recorded frames is 466,597), with each one having an approximate duration of 8s. The root folder “dataset” is divided in ten subfolders (e.g., “subject_1”), with each one containing the data collected by each participant, using the pseudo anonymization IDs depicted in Table 1. Every subject-related folder consists of three subfolders, “env_1”, “env_2” and “new_objects” (i.e., environment 1, environment 2 and environment 3, respectively), which correspond to the environment that the data collection took place. It should be noted that we used “new_objects” naming instead of “env_3” to emphasize the fact that this subset contains different objects compared to the other two places. Inside each environment-related folder there are 5 folders defining the object used during the recording process (i.e., the ground truth label when it comes to machine learning purposes). Finally, the naming convention used for the CSV files defines whether the radar device was moving or was static (only “moving” was considered in RadIOCD since the indoor objects and the participants acting as “human” objects were static) and the timestamp in Unix format (e.g., *moving_1685454309.0061283.csv*). The whole tree-structure of the published dataset is illustrated in Fig. 5.

### Data description

The CSV files recorded by the mmWave radar the contain the details of the generated point clouds. In particular, it contains the following information:

- “Frame #” column, presents the frame ID;
- “# Obj” column, denotes the number of points included in the current frame;
- “X” column, depicts the distance (in meters) between the reflected object and radar on the X-axis. The negative values are at the left of the radar while the positive ones are at the right;
- “Y” column, depicts the distance (in meters) between the reflected object and radar on the Y-axis (contains positive values since the target object is always in front of the radar);
- “Z” column, depicts the distance (in meters) between the reflected object and radar on the Z-axis. The negative values are below the receivers of the radar while the positive ones are above them;
- “Doppler” column, depicts the velocity (in m/s) of the reflected points. The negative values are obtained by surfaces approaching the radar while the positives from surfaces moving away from the sensor;
- “Intensity” column, which shows the power level. It can be converted to dB to measure the intensity of the reflection by using the following equation: 10*log_10_(*power*);
- “Presence” column, which shows for each detected point whether it belongs to an object of interest (value equal to 1) or not (i.e., it belongs to background objects or it is noise) and its value is equal to 0. It should be noted that further processing is needed to define the final points of an object of interest (see next subsection).
- “y,m,d,h,m,s” column, depicts the timestamp in Unix format;
- An example showing few rows of a recorded raw file can be shown in Table 4.

**Table 4:** Sample demonstrating the labels of columns in the CSV files and the corresponding possible values.

Frame #	# Obj	X	Y	Z	Doppler	Intensity	Presence	y,m,d,h,m,s
17300	9.0	−0.017578	0.023438	−0.070312	0.000000	4784.0	0	1683621599
17300	9.0	0.107422	1.638672	0.503906	−0.587584	177.0	1	1683621599
17300	9.0	0.115234	1.792969	0.359375	−0.587584	707.0	1	1683621599
17300	9.0	0.062500	1.980469	−0.123047	−0.587584	382.0	1	1683621599
17300	9.0	−0.609375	3.101562	0.732422	−0.587584	863.0	0	1683621599
17300	9.0	−0.458984	3.003906	−2.048828	−0.587584	896.0	0	1683621599
17300	9.0	3.833984	4.287109	1.021484	−0.293792	161.0	0	1683621599
17300	9.0	−0.042969	0.062500	0.003906	0.000000	4381.0	0	1683621599
17300	9.0	−1.832031	3.044922	0.892578	−0.587584	585.0	0	1683621599

### Processed data

The mmWave sensors are vulnerable to noise, resulting in randomly scattered points and artificial reflections, including clutter and multipath reflections. Even though the used device (i.e., IWR1443Boost) has removed many noise points utilizing the static clutter removal algorithm CA-CFAR, there are still many points produced by high-order reflection between the moving user and static objects^28^; these noise points are particularly pronounced in confined indoor environments where “ghost” objects and multipath rays are likely to manifest^16^. The mitigation of this noise is essential to prevent these “ghost” artifacts from being misinterpreted as false positives in the data processing pipeline. To this end, we adopted the Density-Based Spatial Clustering of Applications with Noise (DBSCAN) algorithm^29^ as the first processing module to remove the noise points in the point cloud. Since DBSCAN does not require the number of clusters to be known beforehand, it possesses a noise rejection capability that, combined with its density-based clustering mechanism, enables effective and automatic separation of noise from distinct objects, and has a relatively low computational complexity, of about $O(n\,\log \,n)$, where *n* is the number of data points^11^.

After applying the DBSCAN to all the examples of dataset, as a second processing step, we manually annotated it frame-wise; i.e., we marked whether the object of interest was detected by the radar in the current time frame, by exploiting the fact that the user is approaching the object in a straight line, so the distance of the user and the identified cluster has to be continuously decreasing. This information is recorded within the CSV files under the “Presence” column. Moreover, in real-world measurements the points of the same object are coherent in the horizontal (X-axis and Y-axis) plane, but more scattered along the vertical (Z) axis^4^, thus, we set the “Presence” value to 0 for points that had extremely high or low Z-axis values. It is also worth noticing that in contrast to radars deployed on robots that move on smooth terrains^16^, motion artifacts are more probable when it comes to wearable devices producing even more noisy point clouds, as depicted in Fig. 3.

Afterwards, exploiting the produced “Presence” column we segmented the collected dataset using a time-window of 1s and overlap 90%, where each example contains 10 frames since the sampling rate of the radar was set to 10Hz. Examples where the object of interest was not present in 80% of the total frames were discarded. Afterwards, we employed again the DBSCAN algorithm to cluster the annotated points but within each time window (i.e., example) this time. As a final processing step to remove noise points, since the participants were moving straight forward towards the selected objects without any other items between them, the 3D points that belong to the closest cluster, having their center of mass less than 0.3m left or right the radar and average absolute doppler velocity value higher than 0, were considered to consist of the object’s point cloud. This final step, has an approximate computational complexity of about $O(K\cdot {n}_{pr}\log {n}_{pr})$, where *K* denotes the produced number of segments, and *n*_*pr*_ is the number of data with “Presense” value equal to 1. In addition to this, we set thresholds on the Y-axis because when the objects were far away from the user (above 2.5m) or closer than 1m in some cases (e.g., backpack) the obtained object points were extremely sparse, and not useful to be processed by a machine learning algorithm. This segmentation and curation process led to the creation of 76,821 1s segments (i.e., a) backpack: 16,027, b) chair: 19,825, c) desk: 6,258, d) human: 21,393 and e) wall: 13,318).

### Metadata

The *metadata.txt* file contains information about: (i) the participants (ID, gender, height, weight and age), (ii) the radar configuration values (frequency, number of Tx antennas, number of Rx antennas, azimuth resolution, range resolution, maximum range, maximum radial velocity, radial velocity resolution, frame duration, range detection threshold, and frame rate), and (iii) the objects’ dimensions (height, width, depth).

## Technical Validation

### Exploratory data analysis

**Fig. 6:**
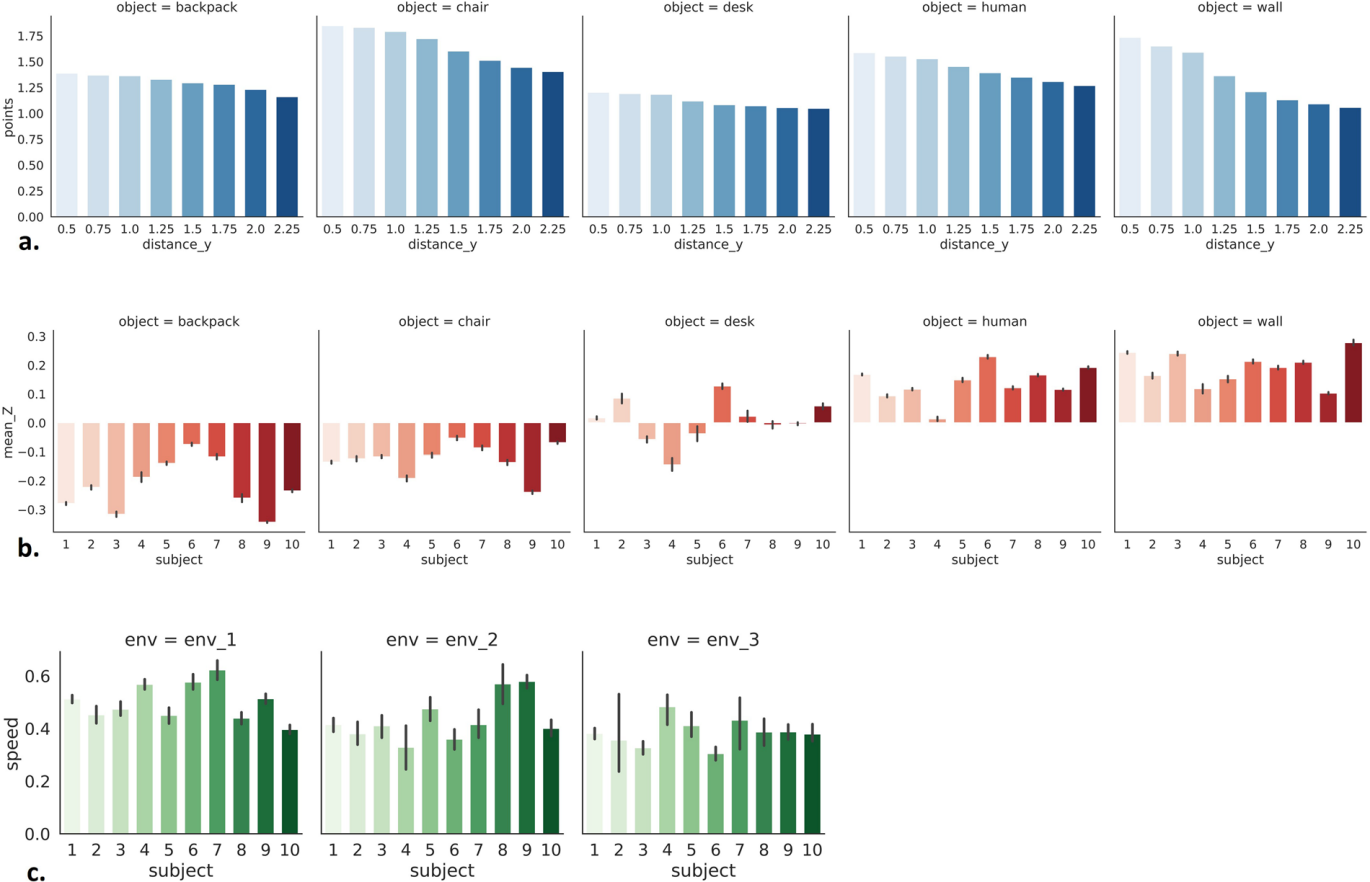
EDA performed on RadIOCD, where (**a**) shows the average number of points created by each object w.r.t. its distance, (**b**) displays the average height of points created by each object w.r.t. the subject wearing the device, and (**c**) presents the average speed of each subject w.r.t. the environment.

In order to understand better the statistical properties of the collected dataset we performed an Exploratory Data Analysis (EDA). The main characteristics are summarized using statistical graphics that present the average number of points created by each object w.r.t. its distance (Fig. 6a), the average height of points created by each object w.r.t. the subject carrying the device (Fig. 6b) and the average speed of each subject w.r.t. the environment (Fig. 6c). As expected, most points are created by objects with larger surfaces, while desk even though it a much larger object than the chair and the backpack, its reflection surface is very small. Moreover, in the following plot (Fig. 6b) we observe that the height of the object is an important feature, where the wall and human produce high values, the desk intermediate, and the chair and backpack small ones. Finally, even though the subjects’ moving speed does not seem to be informative as a feature, it is crucial for the reproducibility of the dataset, providing a perspective of the whole application and the selected time window. The subjects were moving at an average speed of 0.5m/s with most of the objects being detected at around 2.0m, thus the selected 1s window seems to be appropriate to warn the user as soon as possible for the type of the upcoming object. Having larger window sizes such as 3s may have led to better algorithmic performance, but the user would be warned only a few centimeters before reaching the objects, leaving him/her no time to react.

Based on the performed EDA and exploiting the exported clusters, we extracted time-domain features using as input the X, Y, Z coordinates. In particular, we estimated the mean, maximum, minimum and standard deviation for each axis and calculated the total number of points included in each segment, leading to 13 features. All the described processing pipeline and the extracted features can be derived using the corresponding code, along with the object tracked, the subject and the environment where the recording took place, to facilitate the dataset split and processing for machine learning purposes.

### Baseline Machine Learning analysis

We examined 5 different classifiers to check their effectiveness in classifying the detected objects. In particular, we trained the following models: a) Logistic Regression (LR), b) Decision Trees (DT) with maximum depth equal to 20, c) Random Forests (RF) with maximum depth equal to 20 and 30 estimators, d) k-Nearest Neighbors (k-NN) with k equal to 15 and e) a Deep Dense Neural Network (DDNN). For the latter we applied a random search to fine-tune its hyperparameters focusing on the number of neurons per layer and the total number of hidden layers. The final DDNN architecture consists of 4 fully connected hidden layers with the first two having 256 neurons and the final two 128. Each one of them is followed by ReLU (Rectified Linear Unit) activation function and a dropout layer (with 0.1 rate) to increase the model’s robustness to overfitting. The output layer contains 5 neurons outputting the probabilities of each class.

Regarding the dataset split, we evaluated two different approaches, a user dependent 10-fold Cross Validation (CV) and a user independent Leave-One-Subject-Out (LOSO) CV, where the data of 9 subjects are used for training and one for validation and testing, to assess the algorithm’s performance in unseen subjects. As metrics we selected accuracy and f1-score, since the produced dataset is imbalanced having “human” as dominant class. Table 5 shows the 10-fold CV and LOSO CV evaluation of the trained algorithms. The DDNN surpasses the RF performance, which achieves the highest evaluation scores regarding the common ML algorithm, by a significant margin (2.35% in f1-score for the 10-fold CV and 3.0% in f1-score for the LOSO CV). Moreover, as expected, the 10-fold CV reaches higher scores for all the selected algorithms, since data from all the participants are included in this set up during training.

**Table 5:** Evaluation results of the selected baseline algorithms.

Model	10-fold CV		LOSO CV	
	Accuracy %	F1-score %	Accuracy %	F1-score %
LR	67.86	62.27	63.95	57.93
DT	66.47	61.99	59.79	54.78
RF	75.10	70.21	67.72	61.89
k-NN	72.47	67.26	65.28	59.37
DDNN	**76.53**	**72.55**	**70.44**	**64.88**

**Fig. 7:**
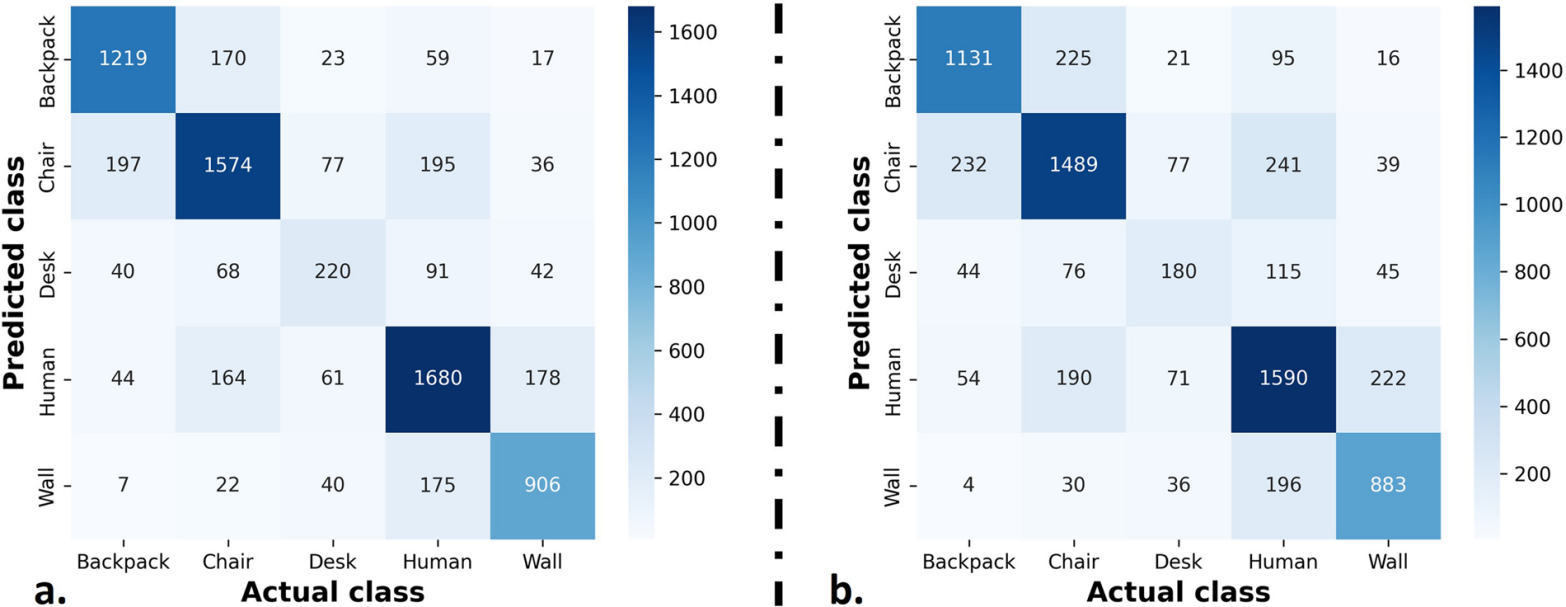
Confusion matrices obtained for the (**a**) 10-fold CV and the (**b**) LOSO CV.

Finally, Fig. 7 displays the confusion matrix of the 10-fold CV. Several “human” examples are misclassified as “wall” and vice versa, since these two are the more dense and tall objects (Fig. 6b). Moreover, the algorithm struggles to distinguish the “backpack” and “chair” classes, since they both represent relatively small objects, while its performance on recognizing “desk” is quite low especially for the LOSO CV case.

### Comparison with public radar-based datasets

In the current section, we encounter point cloud datasets originated by mmWave radars. As aforementioned, although, there exist several LiDAR-based datasets for indoor object classification and segmentation^30,31^, only few datasets rely on mmWave radars, with RadIOCD, to the best of our knowledge, being the first one using a wearable mmWave radar sensor. Due to this lack of existing indoor mmWave datasets, we present also publicly available radar-based datasets that can be exploited for applications related to outdoor object classification, human tracking, and human activity/gesture recognition.

Starting with existing mmWave radar-based point cloud datasets captured in indoor scenarios, milliCap^18^ was recorded using an mmWave radar deployed on a mobile robot and cab be used for object classification. It consists of a training and a test set comprised by 27,952 and 17,583 samples (two test buildings) respectively, while the obtained point clouds represent 5 classes: door, glass, lift, wall, and unknown class (e.g., basins, tables, chairs, sofa and fridges). Another dataset is MilliNoise^16^ that focuses on identifying noise points instead of classifying objects; it consists of the 12M points accurately labeled as true/noise point captured by an mmWave sensor installed on a moving wheeled robot. Similar to RadIOCD, besides the X, Y, Z-axis coordinates, each point’s velocity and intensity information are provided, while each point’s distance to its closest obstacle in the scene is also estimated to allow casting the denoising task into the regression framework. ColoRadar^17^ is another publicly available dataset containing approximately 2 hours recordings of 4D data from two mmWave radar sensors, 3D lidar point clouds, IMU measurements, and groundtruth pose information. ColoRadar’s purpose is to enable robotic mapping and state estimation in highly diverse indoor, outdoor environments instead of object classification.

Regarding automotive datasets, RadarScenes^32^ is a publicly available dataset for automotive radar point cloud perception tasks; it consists of 11 object classes (car, large vehicle, truck, bus, train, bicycle, motorized two-wheeler, pedestrian, pedestrian group, animal, and other) and a total of over 7,000 road users which are manually labeled on 100 km of diverse street scenarios. The dataset offers information such as the X, Y coordinates and the Doppler velocity. NLOS-Radar^33^ contains a total of 100 captured sequences in-the-wild automotive scenes. The authors designed 21 different scenarios, useful for detection, classification, and tracking of hidden object tasks. For the classification task the included classes are background, cyclist and pedestrian. Recently, The TJ4DRadSet, as introduced by Zheng *et al*.^34^, includes 4D radar data points, including range, azimuth, elevation, and Doppler velocity. This dataset was meticulously gathered across diverse driving scenarios and comprises a total of 7,757 synchronized frames distributed across 44 continuous sequences. Each frame is annotated with 3D bounding boxes, tracking IDs, and includes eight distinct classes: cars, buses, trucks, engineering vehicles, pedestrians, motorcyclists, cyclists, and tricyclists. Similarly, VoD^35^ comprises 8,693 frames capturing synchronized and meticulously calibrated LiDAR, camera, and 4D radar data gathered within intricate urban traffic settings. It features a total of 123,106 annotations delineating 3D bounding boxes for various objects, both in motion and stationary. These annotations encompass three primary classes: pedestrians, cyclists, and cars.

In the realm of human activity and gesture recognition, the MMActvity dataset, also referred to as RadHar, stands out as the most widely recognized and benchmarked dataset^13^. An IWR1443BOOST radar was utilized to collect the point clouds mounted on a tripod stand at a height of 1.3m, with a sampling rate equal to 30Hz. In particular, two users performed 5 different activities (walking, jumping, jumping jacks, squats and boxing) in front of the radar. IWR1443BOOST was, also, used in mHomeGES^14^ with the sampling rate was set to 10 frames per second. The participants performed 10 arm gestures within 1.2 meters to 3 meters. The published dataset incorporates 22,000 instances collected from 25 persons. Finally, Pantomime^15^ is another gesture recognition dataset comprised by point clouds that was, also, collected using an IWR1443 radar. 45 subjects participated in the collection performing 21 gestures in five indoor environments (open, office, restaurant, factory and through-wall).

Another application taking advantage of the mmWave radar point clouds is that of people tracking and identification. These applications usually rely on applying DBSCAN to cluster the points over a specified time span and use the Hungarian algorithm and Kalman filters to identify whether the tracked humans in previous *n* frames *f*_*t*−1,*t*−2...*t*−*n*_ are, also, present in each frame *f*_*t*_^4,11^. Unfortunately, even though there is a growing interest by the research community only Meng *et al*.^28^ have made their dataset publicly available. The mmGait dataset was compiled by enlisting the participation of 95 volunteers tasked with navigating through three distinct scenarios across two varied environments. Their movements were recorded using both IWR1443 and IWR6843 radar systems, each operating at a frame rate of 10Hz, while the recordings’ duration is approximately 30 hours.

Finally, it is also worth mentioning datasets relying on Ultra-wideband (UWB) sensors for activity/gesture recognition and human tracking. UWB-gestures^36^ is a public dataset of 12 dynamic hand gestures acquired using impulse UWB radar sensors. The dataset contains 9,600 samples collected from eight human participants, using three radars placed at different locations. OPERAnet^37^ is a multimodal activity recognition dataset obtained using radio frequency, UWB and vision-based sensors. It consists of 8 hours of data collected by 6 participants performing 6 daily in two different rooms. Apart from human activity recognition, OPERAnet can be used for tracking humans in indoor environments. UWB positioning data set contains measurements from four different indoor environments. The data set contains measurements that can be used for range-based positioning evaluation in different indoor environments. For similar purposes, but focusing more on human tracking and less on human activity recognition, UWB dataset was built^38^; this dataset consists of approximately 1.6 hours of annotated measurements collected in a residential environment. Apart from providing the target’s location, the data contain the ground truth for the human activity that was performed, namely, sitting, standing and walking. UWB-based human indoor positioning is, also, the scope of the dataset published by K. Bregar^39^. This dataset contains data acquired by 9 UWB-based measurement nodes (i.e., 8 fixed devices and one mobile positioning device) placed in four different indoor environments.

## Data Availability

The code for downloading, reading, pre-processing and applying the reported baseline machine learning algorithms can be found on GITHUB via the following URL (https://github.com/ounospanas/RadIOCD). The code was written in Python 3.8 and is provided in a *.ipynb* format (https://jupyter.org/). In particular, we used Pandas (https://pandas.pydata.org/) for loading the CSV files, Numpy (https://numpy.org/) library for data pre-processing, segmentation and feature extraction, Scikit-learn (https://scikit-learn.org/) for training the common machine learning algorithms and the Tensorflow (https://tensorflow.org/) framework to develop and train the neural networks.
